# Prenatal Calcification of the Inferior Vena Cava and Renal Veins in a Normal Neonate

**DOI:** 10.1100/tsw.2006.163

**Published:** 2006-06-30

**Authors:** Daniel Ranch, Michael O. Aigbe, Emmanuel C. Gorospe

**Affiliations:** ^1^ Department of Pediatrics, University of Nevada School of Medicine, Las Vegas, USA; ^2^ The Children's Nephrology Clinic, Las Vegas, USA

**Keywords:** calcification, inferior vena cava, renal veins

## Abstract

Prenatal calcification of the inferior vena cava (IVC) and renal veins is a rare condition with unclear etiology and prognosis. It occurs with renal vein thrombosis in utero and is associated with congenital anomalies and abnormal prenatal hemodynamic status. We report a rare case of prenatal IVC and renal vein calcification in a normal neonate without any history of compromised prenatal or perinatal condition, or significant deterioration of kidney function.

## CASE REPORT

A 1-week old male neonate was referred for evaluation of possible nephrolithiasis based on prenatal ultrasound at 28 weeks of gestation, which suggested calcifications in the left kidney. At birth, an abdominal ultrasound confirmed vascular-like calcifications in the left kidney. On review of the medical history, the patient was born full term. There was no history of maternal illness, trauma during pregnancy, or complication at birth.

We requested an abdominal CT scan without contrast, which revealed calcifications in the left renal veins extending to the inferior vena cava (IVC) (Fig. 1). Hematologic parameters, serum electrolytes, blood urea nitrogen, and creatinine levels were all within normal range. Screening for possible prothrombotic factors such as lupus anticoagulant and protein C and S levels was not done in the initial consult. Nevertheless, clotting time, thrombin and prothrombin time remained within normal limits even on subsequent follow-up. Lastly, the patient did not have any clinical evidence of venous obstruction or renal dysfunction.

## DISCUSSION

Prenatal calcification of the renal veins and IVC is a rare event. It is attributed to idiopathic thrombus formation[1] from possible hypotension or hypoxia *in utero*[2]. It is associated with congenital anomalies or compromised hemodynamic status.

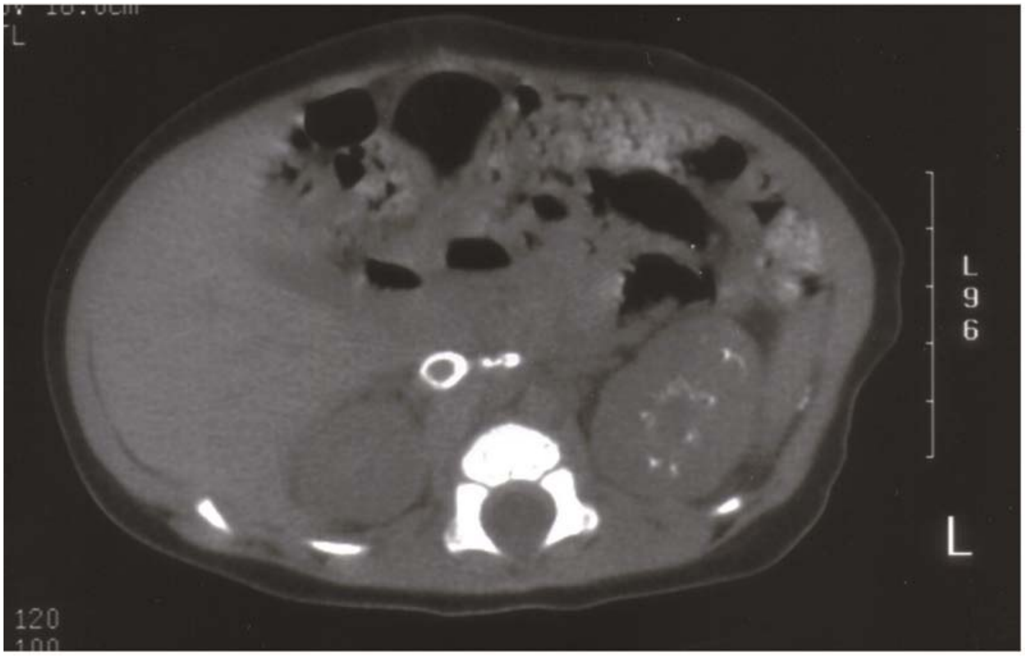

**FIGURE 1.** CT scan of the abdomen presenting ring calcification of the IVC with involvement of the renal veins.

In contrast, postnatal renal venous thrombosis is a common venous thromboembolic event in neonates[3]. It can be the result of postnatal hypercoagulable conditions, sepsis, and iatrogenic causes such as indwelling catheters. The usual calcified IVC thrombus is commonly described as a bullet-shaped radiodensity[4] that differs from the ring-shaped calcification of this case.

There are conflicting reports as to the prognosis of renal venous thrombosis. A series of reports[5,6,7,8] presented decreased renal function and subsequent atrophy of the involved kidney, while others[2,9,10] reported cases with preservation of normal kidney function. The management of renal venous thrombosis is also controversial[2]. Suggestions from experts range from conservative management[11] to invasive thrombectomy and nephrectomy[12].

In this case, continued observation was the treatment of choice as the patient was in good health without any clinical or laboratory manifestations of compromised kidney function. Our experience in this case supports the observation of Wilkinson et al.[2] that suggests that idiopathic prenatal renal venous thrombosis has better prognosis than its postnatal occurrence.
